# The effects of whey protein fibrils on the linear and non-linear rheological properties of a gluten-free dough

**DOI:** 10.3389/fnut.2022.909877

**Published:** 2022-07-29

**Authors:** Shengyue Shan, Da Chen, Enrico Federici, Owen G. Jones, Osvaldo H. Campanella

**Affiliations:** ^1^Department of Food Science and Technology, The Ohio State University, Columbus, OH, United States; ^2^Department of Animal, Veterinary and Food Sciences, University of Idaho, Moscow, ID, United States; ^3^Department of Food Science, Purdue University, West Lafayette, IN, United States; ^4^Whistler Center for Carbohydrate Research, Purdue University, West Lafayette, IN, United States

**Keywords:** gluten-free dough, whey protein fibril, viscoelasticity, rheology, strain-hardening property, cryo-SEM

## Abstract

The increasing awareness of the celiac disease, an autoimmune disorder caused by the consumption of products containing gluten, has led to a growing interest in the development of gluten-free bakery products. In this study, whey protein fibrils (WPFs) were incorporated to mimic the fibrous network of gluten. The rheological properties and microstructure of the developed gluten-free doughs were evaluated and compared with gluten doughs. Protein fibrils were prepared by heating a whey protein isolate (WPI) solution at 80°C in an acidic environment with low salt concentration, and then the fibril lengths were adjusted by leveling up the solution pH to 3.5 and 7. The dimensions of the fibrils were measured by atomic force microscopy (AFM). Rice and potato starches were mixed with fibrils, WPI, gluten, or without protein, to form different doughs for further investigation. Shear tests, including stress sweep, frequency sweep, and creep recovery, were performed to study the viscoelastic properties of doughs under small or large deformation. The strain-hardening properties of doughs under biaxial extension were studied by the lubricated squeezing flow method. The microstructure of the doughs was characterized by cryo-scanning electron microscopy (cryo-SEM). Compared with doughs prepared with WPI and no proteins, doughs incorporating fibrils showed comparable linear viscoelasticity to gluten dough tested with stress sweep, frequency sweep, and creep recovery in the linear viscoelastic region. More differences between the protein fibril doughs were revealed in the rheological properties in the non-linear region. Creep recovery parameters, such as compliance, elastic moduli during the creep, and recovery stages of gluten dough, were like those of WPF pH7 dough, but significantly different from those of the WPF pH3.5 dough. Strain-hardening properties were found in the WPF pH7 dough, although not in WPF pH3.5 dough. Microstructural characterization showed that both fibrils prepared with the different conditions formed a continuous protein phase for the improvement of dough cohesiveness, but the structure of the phase was different between the two fibrils. To summarize, whey protein fibril at pH 7 seemed to have the potential of being used as an ingredient with similar functions to gluten in gluten-free bakery products.

## Introduction

Over the past few decades, the market for gluten-free products has expanded remarkably due to the growing awareness of the celiac disease (CD). CD is an autoimmune disorder caused by gluten ingestion in persons who are genetically susceptible (1). Approximately, 1–2% of the general population is affected by CD, although some may remain underdiagnosed worldwide according to population studies (2, 3). Currently, there is no effective therapeutic treatment available for CD except for strict implementation of a lifetime gluten-free diet (1, 4).

Despite causing an inflammatory response in CD patients, gluten, which is generally defined as a mixture of storage proteins in wheat, contributes to the unique viscoelasticity of dough, and is critical for the baking performance of leavened bakery products (5–7). Based on the solubility in aqueous ethanol, gluten proteins can be categorized into glutenin and gliadin (7). With energy input from mixing, hydrated gluten forms a three-dimensional network possessing viscoelastic properties, where glutenin is responsible for dough cohesiveness and elasticity, and gliadin contributes mainly to the viscosity of dough (8, 9). The elimination of gluten in gluten-free bread production has introduced technical challenges in developing products with similar quality and acceptability to gluten-containing products (10). Due to the lack of a gluten network, gluten-free bread may exhibit problems, such as unsatisfactory crumb and crust characteristics, dry mouthfeel, reduced volume, lack of cell structure, and short shelf-life (11, 12). The product quality and breadmaking process are essentially related to the dough’s rheological properties, and it is necessary to study the linear and non-linear viscoelasticity of doughs for production optimization. Linear viscoelasticity is reflected as the response of a material to a sufficiently small deformation, whereas non-linear viscoelasticity occurs under a much larger deformation. Extensive studies have been conducted on the linear viscoelasticity of doughs, especially with the small amplitude oscillatory shear (SAOS) technique (13–17). However, in practical production, doughs are usually subjected to not only large deformation, but also deformation induced by different types of strains besides shear, including compression, extension, and torsion (18). Despite the difficulties in the data collection, analysis, and interpretation, the study of non-linear viscoelastic properties of dough is more closely related to its behavior in processing and performance of the final product (19). The strain-hardening property of dough under extension, one of the non-linear properties, has been reported to be an effective indicator of dough baking quality (20, 21). Fundamental studies of linear and non-linear rheological properties could provide guidance and insights into the development of high-quality gluten-free doughs.

Attempts have been made to improve the quality of gluten-free bread, and one of the promising approaches has been to incorporate functional ingredients, which are capable of forming or promoting a network structure in the product (10). The potential of hydrocolloids, non-gluten proteins, and enzymes to act as gluten replacers has been investigated previously (12, 22–24). Proteins from other food resources, especially dairy, are often added to the gluten-free bread formula for improving the quality attributes of the product. Dairy proteins are capable of forming a network similar to that of gluten (25). It has also been reported that the function of dairy proteins in a gluten-free dough system could be improved with enzymatic treatment and structural modification. For instance, transglutaminase was found to be able to promote the formation of a stable protein network by catalyzing cross-linking reactions among proteins from skim milk in gluten-free bread (26). In another study, whey protein was structured into mesoscopic particles (length 100 nm–100 μm) and developed into a gluten-free dough, and the dough showed better proofing and baking performance than those prepared with unstructured whey protein (27).

Protein fibrillization is a strategy to improve the functionalities of proteins that has gained an increasing interest in food science research (28, 29). Protein fibrils, or fibrillar proteins, are highly ordered, self-assembled protein aggregates (30). Protein fibrils can be formed with globular proteins from various food resources, such as pea, egg, soy, dairy, and so on (31). As one of the most widely used ingredients in the food industry, whey protein isolate (WPI) has also been commonly used in the preparation of protein fibrils, essentially due to its economic viability (32). Whey protein fibrils usually have a length of several micrometers and a height of a few nanometers (33, 34). They often display prominent mechanical properties, including semi-flexibility, high elasticity, high resistance to deformation, and high stiffness with Young’s modulus in the range of 2–4 GPa (28, 35). Additionally, whey protein fibrils show ideal surface properties (35, 36) for reducing the surface tension of air bubbles to act as foam stabilizers (37). It was shown previously that whey protein fibril at low pH could interact with potato starch and increase the elasticity of a potato starch gel (38). The physicochemical properties of whey protein fibril make it a promising replacement of gluten, which forms a viscoelastic matrix able to stabilize gas bubbles and embed starch granules. The pH of foods generally falls in the range of 4–7, thus for the application in food, it is necessary to consider the effects of pH on the morphology, stability, and functionality of the whey protein fibril (28, 39). More studies are required to investigate the behavior of whey protein fibrils under different pH levels in a food system.

The present study aimed at providing insights into the effects of whey protein fibril (WPF) on the fundamental rheological properties and microstructure of a gluten-free dough system. WPFs were prepared by heating a WPI solution in a low pH, low ionic strength environment, and further adjusting pH to 3.5 or 7, respectively. WPFs at different pH values were later incorporated into starch-based model systems and compared with systems developed with WPI, gluten, or without protein through fundamental rheological tests and microstructural characterization. Rheological shear tests, including stress amplitude sweep, frequency sweep, and creep recovery, were utilized to study both the linear and non-linear viscoelasticity properties of doughs. The strain-hardening behavior of dough was studied with a biaxial extensional test, namely the lubricated squeezing flow method. Cryo-scanning electron microscopy (cryo-SEM) was utilized to investigate the structure of doughs. The objective of the study was to demonstrate the feasibility of applying WPF as a novel gluten replacer in the development of a gluten-free dough with comparable rheological properties to wheat gluten dough.

## Materials and methods

### Materials

Whey protein isolate (Hilmar 9400) was kindly donated by Hilmar Ingredients (Hilmar, CA, United States) with 93.4% protein (dry basis), 4% moisture content, 0.6% fat, and 2.6% ash. Native potato and rice starch (CAS 9005-25-8) were purchased from Bob’s Red Mill (Milwaukie, OR, United States) and Sigma-Aldrich (St. Louis, MO, United States), respectively. The gluten from wheat (CAS 8002-80-0, TCI America, Portland, OR, United States) and sodium chloride (CAS 7647-14-5) were procured from Thermo Fisher Scientific (Waltham, MA, United States). Food-grade granular sugar (Kroger, Cincinnati, OH, United States) was purchased from a local grocery store. Deionized water was used in the dough preparation.

### Preparation of whey protein fibril

Whey protein isolate fibrils were prepared as described in a previous study (38) with minor modifications. Briefly, the WPI solution (2.5%, w/v) was acidified to pH 2 with 6 M HCl, and then heated and stirred (∼100 rpm) in an oil bath at 80°C for 12 h. The solution was then cooled in an ice-water bath, adjusted to pH 3.5 or 7 with 1 N or 3 N NaOH, and freeze-dried for further use.

### WPF characterization

The morphology of the fibrils was characterized with an MFP-3D Atomic Force Microscope (AFM, Asylum Research, Oxford Instruments, United Kingdom) under intermittent-contact mode using an aAC240TS probe (2 N/m, Olympus Instruments, Japan). A diluted WPI fibril solution (protein content 0.1-0.2 mg/mL) was loaded to freshly cleaved mica, water-rinsed after 2 min, and then dried in air for further observation. The contour length of more than 150 fibrils in each sample was measured by image analysis using ImageJ (National Institutes of Health, Bethesda, MD, United States).

### Development of the doughs for investigation

The moisture content of the prepared doughs was maintained at 41% by varying the water added to the formula. For the dough sample prepared without protein, the dry powder was composed of 46.7% (w/w) rice starch, 46.7% (w/w) potato starch, 1.9% (w/w) salt, and 4.7% (w/w) sugar. For the dough samples with proteins, 42.7% (w/w) rice starch, 42.7% (w/w) potato starch, 8.5% (w/w) protein powder, 1.7% (w/w) salt, and 4.3% (w/w) sugar were mixed as the dry ingredients. The water added to 100 g of dry powder was 46.1 g for dough without proteins, 48.3 g for the WPI dough, 47.8 g for the gluten dough, 48.3 g for the dough prepared with WPF at pH 3.5, and 48.0 g for the dough with WPF pH7.

A stand mixer (model Professional 600, Kitchen-Aid, Benton Harbor, MI, United States) was used in the preparation of dough according to the previously described method. The ingredients were mixed at a power level 2 with a dough paddle attachment for 3 min. The prepared samples were sealed in a polyethylene bag to prevent moisture loss until further use.

### Shear tests

The shear tests were performed at room temperature on a controlled-stress rheometer (model Discovery Hybrid Rheometer 30, TA Instruments, New Castle, DE, United States) equipped with 40-mm diameter cross-hatched stainless steel upper and lower plates to prevent slippage during tests. Approximately, 3.5 g of dough sample was loaded, and the gap size was set to 1.6 mm. The excess sample over the boundary of the geometry was carefully removed with a spatula. The sample was allowed to equilibrate for 120 s before measurements. A solvent trap was used to reduce moisture loss of samples during tests. At least four replicates were performed on each type of sample in each test.

#### Oscillatory stress amplitude sweep

A stress sweep was performed with the stress ranging from 1 to 1,000 Pa at a frequency of 1 Hz to determine the linear viscoelastic region (LVR) of the dough samples. The storage modulus (G’), phase angle (δ), and oscillation strain are reported as a function of stress and compared. The elastic limit yield stress, which is the endpoint of the LVR, is the point where the storage modulus starts to deviate significantly from an LVR plateau (40). Beyond the LVR is the non-linear viscoelastic region (NLVR) where the material is often subjected to large deformations and structural damage may occur. The critical strain corresponding to the elastic limit yield stress was identified for the selection of testing conditions of frequency sweep, and stresses within and beyond the LVR were identified for selecting conditions for studying the linear and non-linear viscoelastic behavior of the samples in the creep recovery tests.

#### Oscillatory frequency sweep

A frequency sweep test was conducted at a stress of 5 Pa (within the LVR) with frequencies ranging from 0.1 to 10 Hz. The results of the frequency sweep were expressed in terms of G* and δ over the frequency range. The viscoelastic behavior of the samples at this amplitude stress was considered linear.

#### Creep recovery test

Creep recovery tests were performed on gluten, WPF pH3.5, and WPF pH7 doughs under shear stress at 5 and 200 Pa, which are representative stresses in the LVR and NLVR. It was not possible to identify stress in the NLVR to study the creep recovery properties for all five dough types. Therefore, the WPI dough and dough without proteins were eliminated from the creep recovery test to keep the study focused on assessing the functionality of WPFs as gluten replacers. The test was composed of creep and recovery stages. During the creep stage, constant shear stress was applied for 300 s. The removal of the constant stress signaled the start of the recovery stage, and then the sample was allowed to recover for 300 s. The strain of the sample was recorded for a total of 600 s, and the data were further analyzed with a fractional calculus model as shown below (41):


(1) $$J\,\left(t\right)=\frac{\epsilon \,\left(t\right)}{\sigma_{0}}=\frac{1}{\Gamma \,\left(\alpha +1\right)}\,\left(\lambda_{1}\,t^{\alpha }\,H\,\left(t\right)-\lambda_{2}\,{\left(t-t_{m}\right)}^{\alpha }\,H\,\left(t-t_{m}\right)\right)$$

Where *J*(*t*) is the compliance of the material (Pa^–1^), ε(*t*) is the strain of the material measured during the test time, σ_0_ is the constant stress applied during the creep stage (Pa), *t* is the test time (s), and *t*_*m*_ is the time when the constant stress is removed. Γ is the gamma function, and H(t) is the Heaviside function defined as below:


(2) $$H\,\left(t\right)=\left\{ \begin{array}{c} 0,\text{if}\,t<0\\ 1,\text{if}\,t\geq 0 \end{array}\right.$$

The order of fractional derivative, α, ranges from 0 to 1, and a small value of α indicates a high material elasticity. Parameters λ_1_ and λ_2_ are defined as the inverse of the creep elastic modulus and recovery elastic modulus of the material, respectively. The values of α, λ_1_, and λ_2_ of the different doughs investigated in the study are reported and compared.

### Biaxial extension test by lubricated squeezing flow test

The biaxial extension is one of the major forms of deformation that the dough is subjected to during fermentation and baking. The dough matrix surrounding a gas cell during its growth is often extended biaxially, resulting in the increase of biaxial stress that could further retard the gas cell growth (42). In this study, a rheological technique named lubricated squeezing flow viscometry was applied to study the properties of the samples under biaxial extension. In the test, the sample is compressed horizontally between two parallel plates under lubricated conditions, and the contact between the sample and plates is considered frictionless (43). The test was performed on gluten, WPF pH3.5, and WPF pH7 doughs on a TA.XT Plus texture analyzer (Texture Technologies Corp., Hamilton, MA, United States) with a 50-kg load cell. The texture analyzer was equipped with Teflon upper and bottom plates of diameter 25.4 mm. The sample was shaped into a cylinder with a diameter of 25.4 mm and a height of 2 ∼ 2.5 mm and then loaded between the plates. Both the plates and the sample were lubricated with a PTFE-based lubricant (3-IN-ONE, Budd Lake, NJ, United States). The samples were compressed to a total engineering strain of 90% by applying constant upper plate velocities of 0.05, 0.1, and 0.2 mm/s. The Hencky strain, biaxial strain rate, stress, and extensional viscosity of the samples were calculated according to the following equations:


(3) $$\begin{array}{c} \epsilon_{H}=\ln\left(\frac{L_{0}}{L_{t}}\right)\\ {\dot{\epsilon }}_{b}=\frac{\text{d}\,\epsilon_{H}}{2\,\text{d}\,t}\\ \sigma =\frac{F}{A}\\ \eta_{\text{ext}}=\frac{\sigma }{{\bar{\epsilon }}_{b}} \end{array}$$

Where *ε_*H*_* is Hencky strain, *L*_0_ is the original height of the sample (mm), *L*_*t*_ is the height of the sample at any time during the test (mm), $\dot{\varepsilon }$_*b*_ is the biaxial extension rate (s^–1^), σ is stress applied in the tests (Pa), *F* is the compression force (N), *A* is the area of the plate (m^2^), and η_ext_ is the extensional viscosity (Pa s^–1^).

### Cryo-scanning electron microscopy

The micrographs of the structures formed by proteins in the doughs were obtained on a field emission scanning electron microscope (FEI Nova NanoSEM, Hillsboro, OR, United States) with an Everhart Thornley detector. A cryo system (GATAN Alto 2500, Pleasanton, CA, United States) was set at −185°C for cryo imaging. The dough samples were mounted on a stub, flash-frozen by immersion in liquid nitrogen slush, and transferred to the cryo preparation chamber of the cryo system. The samples were then cryotomed and immediately transferred to the cryo stage connected to the microscope stage. After sublimation of the ice at −90°C, the samples were sputter-coated with platinum for 120 s in the cryo preparation chamber. The coated samples were imaged at −140°C.

### Statistical analysis

One-way or two-way analysis of variance (ANOVA) was performed with RStudio (R version 4.0.2, RStudio, PBC, Boston, MA, United States) for the statistical comparison of experimental results. Tukey’s HSD method was applied in the *post hoc* test for pairwise comparisons.

## Results and discussion

### Morphology of whey protein fibrils

**FIGURE 1:**
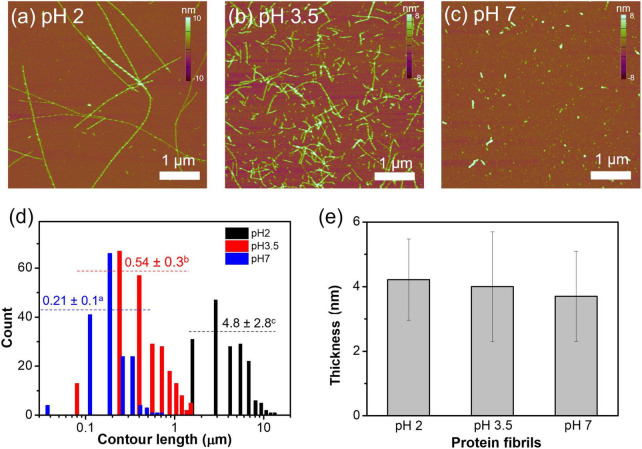
Morphological characteristics of whey protein fibrils at pH 2, 3.5, or 7. **(a–c)** AFM micrographs of whey protein fibrils at pH 2, 3.5, and 7. Fibril heights are denoted in the color bars on the right of each graph. **(d)** Contour length distribution of whey protein fibrils at different pH levels. The number above the dotted line represents mean ± standard deviation of the fibril contour length, and the different letters on the top right represent that the values are significantly different. **(e)** The mean and standard deviation of the thickness of protein fibrils prepared at different pH levels.

The AFM micrographs of WPFs at pH 2, 3.5, and 7 are shown in Figures 1a–c. At pH 2, WPFs were observed to be long and linear strands with an average contour length of ∼ 4.8 μm. When pH was increased to 3.5, the WPFs were largely shortened with an average contour length of ∼0.54 μm. Further increase of pH to 7 degraded the fibrils into smaller fragments (∼0.21 μm contour length). The difference in contour lengths of fibrils under different pH levels was significant, supported by one-way ANOVA (Figure 1d), and similar observations have been reported elsewhere (31, 44). But no significant difference was found among the thicknesses of fibrils across different pH values (Figure 1e), indicating that degradation of WPFs occurred mainly in the longitudinal direction.

When heated at 80°C under pH 2, whey proteins may be molten or partially unfolded because of the repulsive force existing within the protein molecules, and in the meantime, protonation of the carbonyl oxygen promoted by the acidic environment could dissociate the peptide bonds (28). Either condition could promote the formation of long fibrils. The shortened fibril lengths may be caused by the altered electrostatic interaction and protonation status of the fibrils (31, 45). The average thicknesses of WPFs in this study were around 4 nm, similar to the previously reported average thickness of fibrils formed by β-lactoglobulin (β-lg), which is the most abundant protein in WPI. Other whey proteins, such as α-lactalbumin and bovine serum albumin, may not directly form fibrils but could affect the fibrillization mechanism of β-lg, which may explain the large variabilities in the fibril thickness (33, 39, 46).

### Dough development

**FIGURE 2:**
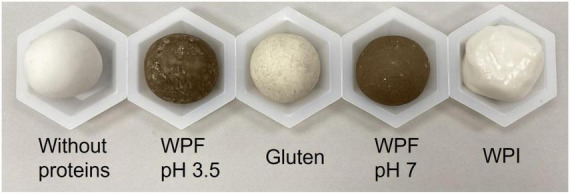
Photographs of the dough samples. Without proteins: no protein was added to the dough; WPF pH 3.5, Gluten, WPF pH 7, WPI: doughs with WPF at pH3.5, gluten, WPF at pH 7 and WPI incorporated as the protein source.

Attempts were made to measure the optimal water absorption of each dough formula using a mixograph (data not shown). However, it was not possible to obtain doughs with similar resistance to mixing or consistency for identifying the optimum amount of water to be added with the mixograph. In the end, the doughs were prepared keeping the moisture content of final dough at 41%, which allowed the comparison of the effects of different proteins on the rheological properties of dough. Therefore, the water amount used to prepare a dough with the same moisture content was chosen for dough preparation to compare the rheological behavior of the dough samples. The photographs of representative samples of each dough type are shown in Figure 2. Doughs prepared with gluten, WPF pH3.5, WPF pH7, and without proteins could form a regular shape during mixing and kneading, but the WPI dough was more similar to liquid and difficult to maintain a fixed shape.

### Identification of linear viscoelastic region

**FIGURE 3:**
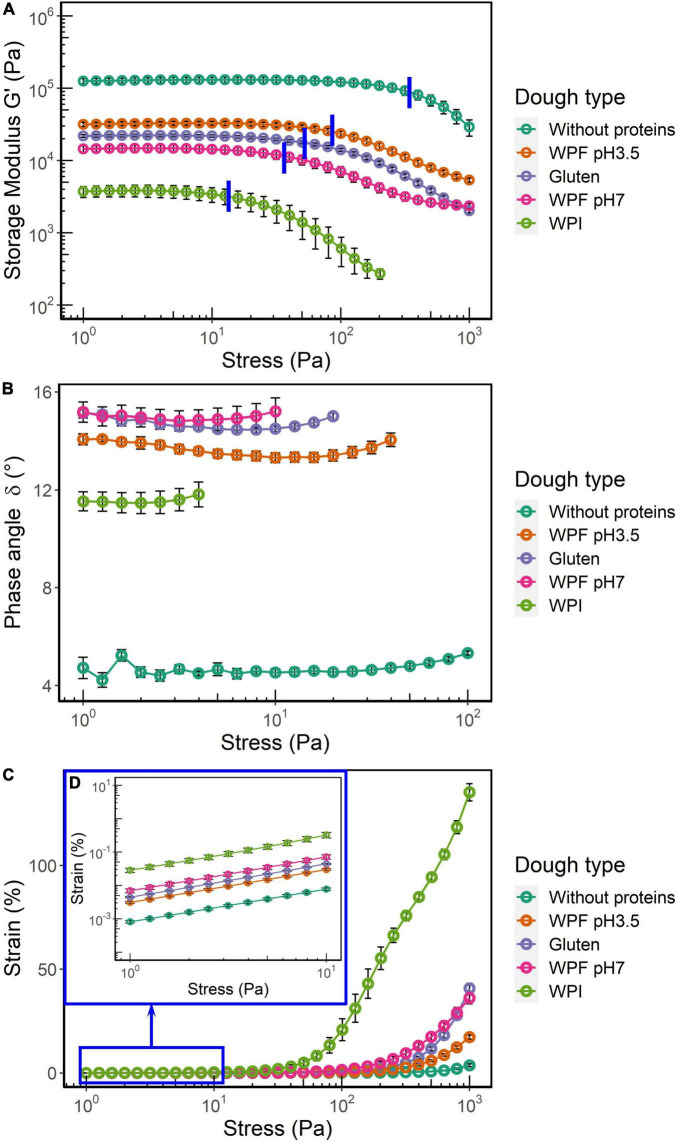
Storage modulus (G’), phase angle (δ), and strain as functions of the applied shear stress. The error bars represent the standard error. **(A)** G’ over the range of stress values. The average values of LVRE were highlighted by blue lines. **(B)** δ within the LVR. **(C)** Strain over the test range of stress. **(D)** Inset for the data of strain when the range of applied stress was 1–10 Pa.

The rheometer used in this study was a controlled stress rheometer; therefore, a stress amplitude sweep test was applied to identify the linear viscoelastic region (LVR). The storage modulus (*G*’), phase angle (δ), and oscillation strain over an oscillation stress range of 1–1,000 Pa are shown in Figures 3A–D. This test provided information that is used for the selection of the testing conditions for the following frequency sweep and creep recovery tests.

*G*’ represents the elastic response of a viscoelastic material subjected to deformation. The plateau of *G*’ in the region of lower stress plotted as a function of stress or strain is usually defined as the LVR, where the deformation is considered as viscoelastic and reversible. In this study, the end of LVR (LVRE) was defined as the stress where the magnitude of storage modulus decreased by more than 3%. The average LVRE values are highlighted by blue lines in Figure 3A. For both *G*’ and LVRE, the dough prepared without proteins had the highest values, followed by the doughs prepared with WPF pH3.5, gluten, and WPF pH7 (*p* > 0.05), while the lowest values were found in the WPI dough. This indicated that the WPI dough had the lowest stiffness and the weakest structure, whereas the highest stiffness and strongest structure were observed in the dough prepared without proteins.

The phase angle δ within the LVR of each dough type is illustrated in Figure 3B. The average δ value of the dough without protein was significantly different from the other doughs. Although the δ value of WPF pH3.5 dough seemed to be slightly lower than WPF pH7 and gluten doughs from the figure, there were no significant differences among the average δ value of these dough types (*p* > 0.05). The δ of WPI dough was significantly lower than that of the gluten, WPF pH3.5, and WPF pH7 doughs, which may be interpreted as the WPI doughs show more elastic, solid-like behavior than the three dough types. This seems to contradict the results of *G*’. It has been noted that *G*’ value is not only related to δ but also to strain as demonstrated by the equation below (47):


(4) $$G^{\prime }\,\left(\omega \right)=\frac{\sigma_{0}}{\gamma_{0}}\,\cos\left(\delta \right)$$

*G*’ is storage modulus, ω is frequency, σ_0_ is the amplitude of the applied stress, γ_0_ is the amplitude of the resulting strain, and δ is the phase angle. The strains of the doughs during the stress sweep are reported in Figures 3C,D. The strain of WPI dough was remarkably higher than that of the other dough types, and showed a linear increase when oscillatory stress was higher than 50 Pa. Even in the low stress region (Figure 3D), the strain was at least five times larger than that of the other dough types. For the stress sweep, ω and σ_0_ were controlled, and a smaller γ_0_ value results in a higher *G*’ value despite the value of δ. Results in the measured strains also could reflect the differences in the doughs from another perspective. For instance, the strain of the dough prepared without proteins with stress of 1,000 Pa was ∼3.7%, indicating that the structure of the dough was nearly intact during the stress sweep and only a slight deformation was observed. The strain of gluten and WPF pH7 in the high stress region, which is beyond the LVR, showed a similar trend and magnitude, whereas lower values were found in the WPF pH3.5 dough. This may indicate that WPF pH3.5 dough has a stronger structure than the other two dough types, but confirmation through separate non-linear viscoelastic region (NLVR) tests is required to validate the statement, since imperfections could be introduced to the material sinusoidal response in the NLVR, and the data in the NLVR from a stress sweep cannot be used for comparison directly (48–50).

In comparison with the dough without proteins, the incorporation of protein into a starch-based dough system greatly decreased *G*’ and increased δ values. Compared to the dough prepared with native WPI, the doughs with protein fibrils exhibited a viscoelastic behavior closer to that of gluten dough in terms of *G*’ and δ. However, previous findings on wheat doughs have shown that the linear viscoelasticity of doughs with various baking performances may be similar because the small deformation used in the tests does not resemble deformations applied during dough handling **(51, 52)**.

### The linear viscoelastic behavior of doughs in the frequency sweep

**FIGURE 4:**
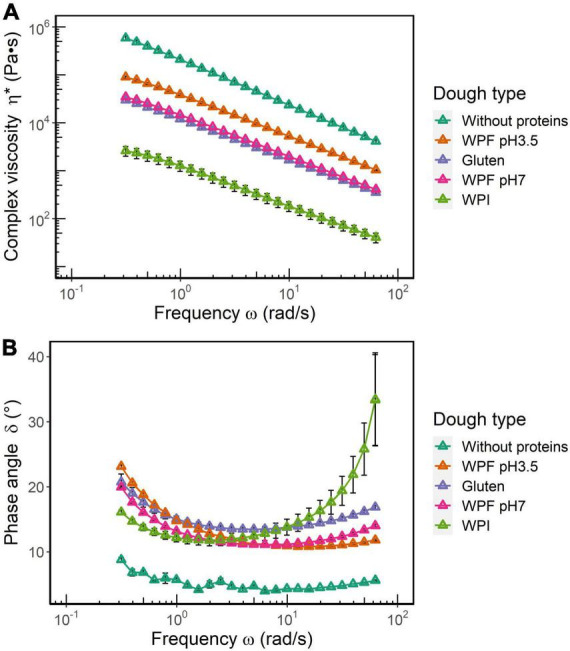
**(A)** Complex viscosity (η*) and **(B)** phase angle (δ) as functions of oscillatory frequency. The error bars represent the standard error.


A comparison of complex viscosity (η*) and phase angle (δ) for all the dough types is illustrated in Figure 4. It is shown that η* values decrease with an increase in frequency, which is typical behavior of a viscoelastic material. Overall, η* of dough without proteins >WPF pH3.5 dough >gluten dough ≈ WPF pH7 dough >WPI dough (Figure 4A). According to a two-way ANOVA, there was no significant difference between η* values of gluten and WPF pH7 doughs, while differences among other dough types were significant (*p* ≤ 0.05). The η* versus frequency relationship indicated that all the dough types weakened at higher frequencies, which may be related to the loss of elasticity. Higher η* values may indicate a very elastic-like behavior, but contrarily, the dough prepared without proteins is considered as a highly viscous material that did not resemble the properties of dough. On the other hand, values of η* for the WPI dough were the lowest, which would be indicating a type of dough with very little elasticity, which is confirmed by the results reflected in the stress sweep (53), and the definition of η* by the equation below:

(5) $$\eta^{{}^{*}}=\frac{\sqrt{G^{\prime ,2}+G\,{"}^{2}}}{\omega }$$

As shown in Figure 4B, the dough without proteins showed the lowest δ (<10°) over the entire frequency range, and it was relatively independent of the frequency, which is characteristic of a true solid material. The δ values of WPI dough were lower than those of gluten, WPF pH3.5, and WPF pH7 doughs in the low frequency range. However, δ values of WPI dough increased rapidly after 2 rad/s, and then became not only higher than the other doughs, but also showed a higher variability after 10 rad/s. The difference in the δ values of the different doughs is likely to indicate that different mechanisms could be attributed to the dough microstructural disruption induced by frequency. Similar results have been observed in systems containing cross-linked protein filaments. The behavior has been attributed to the formation of bundles of cross-linkers, and higher frequencies make these bundles slip past each other (54), thus increasing a more fluid character of the material. Hence for WPI dough, higher frequencies may introduce structural damage to a greater extent, and according to a previous study (55), the addition of WPI to a WPF solution could lead to the decreased viscosity of the solution. Similar softening effects of WPI have been reported in other systems, such as a 3D-printed lemon mousse and a protein paste (56, 57). As reported in section “Preparation and characterization of whey protein isolate fibril,” the contour length of WPF pH3.5 was larger than that of WPF pH7, which may result in higher resistance to frequency-induced deformation in a starch-based dough system.

In the dough without proteins, the rigidity of native starch granules is intrinsically high, and the presence of abundant hydroxyl groups in the starch molecules creates a hydrophilic environment that could interact with water to form a compact and rigid structure with little dependence on frequency as that shown by typical true solids (58, 59). In the high-frequency region, the rigid structure disintegrated because of the lack of a viscous environment, and the fractured dough structure of pure starch would not necessarily flow like a semiliquid viscoelastic dough, as indicated by the low δ at high frequency. Due to the lack of viscoelastic properties suitable to form doughs, further tests using this system were not performed.

### Creep recovery in the linear and non-linear viscoelastic regions

**FIGURE 5:**
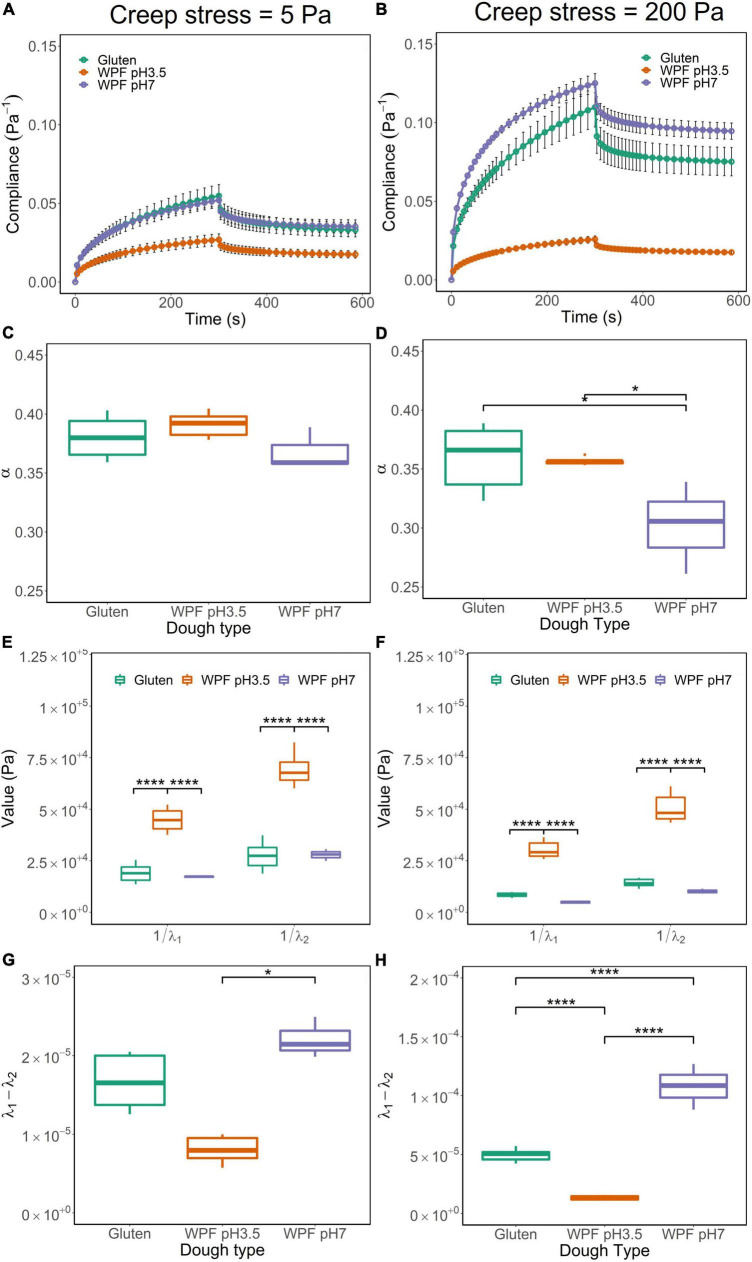
Creep-recovery curves, parameters from the fitted model, including α, the inverse of λ_1_ and λ_2_, and the difference between λ_1_ and λ_2_ for gluten, WPF pH3.5, and WPF pH7 doughs under creep stress at 5 Pa **(A,C,E,G)** and 200 Pa **(B,D,F,H)**. The error bar represents the standard error. Asterisks (*) indicate the significant difference between values (*p ≤ 0.05, ****p ≤ 0.0001).

Doughs with suitable viscoelastic properties were selected for these tests. Therefore, creep recovery tests were performed on the doughs (including gluten, WPF pH3.5, and WPF pH7) with creep stress at 5 and 200 Pa in the LVR and NLVR, respectively. The creep-recovery curves of the doughs are illustrated in Figures 5A,B where the compliance during creep and recovery stages are observed. Creep compliance quantitatively demonstrates the flow capacity of material under the instantaneous application of constant stress and allows the comparison of creep recovery behavior under different stress conditions (60). The doughs showed similar viscoelastic performance, but the maximum creep compliance of both the WPF pH7 and gluten doughs increased when creep stress increased from 5 to 200 Pa, while negligible changes were observed in the WPF pH3.5 dough. The difference in the maximum compliance could be related to the relationship between stress and strain demonstrated by the stress sweep, as shown in Figures 3C,D.

The creep-recovery curves were fitted with a fractional calculus model described in section “Creep recovery test,” and the model showed a good fit with a root mean square error (RMSE) ranging from 1.92 × 10^–6^ to 5.83 × 10^–5^ across all the fittings. As discussed, the model parameters α, λ_1_, and λ_2_ could be interpreted in terms of the rheological properties of the doughs. Two-way ANOVA was applied to statistically study the effects of dough type and creep stress on the parameters.

The shape of the creep stage is illustrated by the parameter α, which indicates the degree of elasticity of a material. Theoretically, α = 0 applies to a purely elastic material and α = 1 to a purely viscous material, and intermediate values correspond to viscoelastic materials with different degrees of elasticity. The α values of the three tested doughs under 5 and 200 Pa are shown in Figures 5C,D, respectively. Overall values were smaller than 0.45, indicating that the doughs were viscoelastic materials with a high elastic behavior.

There was no significant difference in the values of α tested at 5 and 200 Pa for gluten dough, but they were significantly different for WPF pH3.5 and WPF pH7 doughs (*p* ≤ 0.05), which exhibited more elastic behavior at higher creep stress. There was no significant difference in values of α among doughs tested at 5 Pa. However, for 200 Pa stress, the α value of WPF pH7 dough was significantly lower than those of gluten and WPF pH3.5 doughs (*p* ≤ 0.05), indicating that WPF pH7 dough deformed more rapidly upon the application of high and instantaneous stress than the other two dough types.

The inverse of the parameters λ_1_ and λ_2_ are related to the elastic modulus of the samples after creep and recovery, respectively. In other words, the parameters describe the resistance to deformation, during the creep and recovery stages. Thus, the values of 1/λ_1_ and 1/λ_2_, representing the creep and recovery elastic modulus, respectively, are illustrated in Figures 5E,F. In general, both 1/λ_1_ and 1/λ_2_ were smaller when the applied stress was 5 Pa than when the applied stress was 200 Pa for each dough type, and the difference was significant (*p* ≤ 0.05). This indicates that the doughs deformed and recovered faster in the NLVR. In both LVR and NLVR, values of 1/λ_1_ and 1/λ_2_ of WPF pH3.5 dough were significantly different than those of gluten and WPF pH7 doughs (*p* ≤ 0.0001), while there was no significant difference between the latter two. The result implied that WPF pH3.5 dough was more resistant to deformation inducted by creep and recovery.

As a measure of the material deformation path during creep and recovery, the difference (λ_1_ – λ_2_) could be used as an indicator of the recovery capacity of the material. The difference between λ_1_ and λ_2_ is usually small, but when the deformation is permanent, that is, λ_1_ > λ_2_, as illustrated in Figures 5G,H, although the values of (λ_1_ – λ_2_) were rather small with magnitudes of 10^–5^ to 10^–4^ under both creep stresses, the differences in (λ_1_ – λ_2_) between LVR and NLVR were significantly higher in gluten and WPF pH7 doughs (*p* ≤ 0.05), indicating a loss of recovery capacity of these doughs under an applied high stress, while the effect of stress was not significant for the WPF pH3.5 dough. The (λ_1_ – λ_2_) value of WPF pH7 dough was higher than the other two types of doughs in both LVR and NLVR, which indicates a lower recoverability of WPF pH7 dough, and in the meantime, WPF pH3.5 showed high recoverability in comparison to the other two dough types, which may indicate that a weaker structure of WPF pH7 dough is more susceptible to damage.

When the creep stress was 5 Pa, which falls within the LVR for all three types of doughs, the similarities in the creep recovery properties of doughs could be correlated to the properties obtained in the oscillatory tests, as discussed in sections “Oscillatory stress amplitude sweep” and “Oscillatory frequency sweep.” Nevertheless, the non-linear creep recovery behavior of the doughs could better differentiate among the doughs and may show greater potential in predicting dough baking performance, as indicated by previous studies on gluten doughs, and the interactions between proteins could be more dominant in determining the rheological properties of doughs at large deformations (61–63).

### Strain-hardening properties of doughs measured by the lubricated squeezing flow method

**FIGURE 6:**
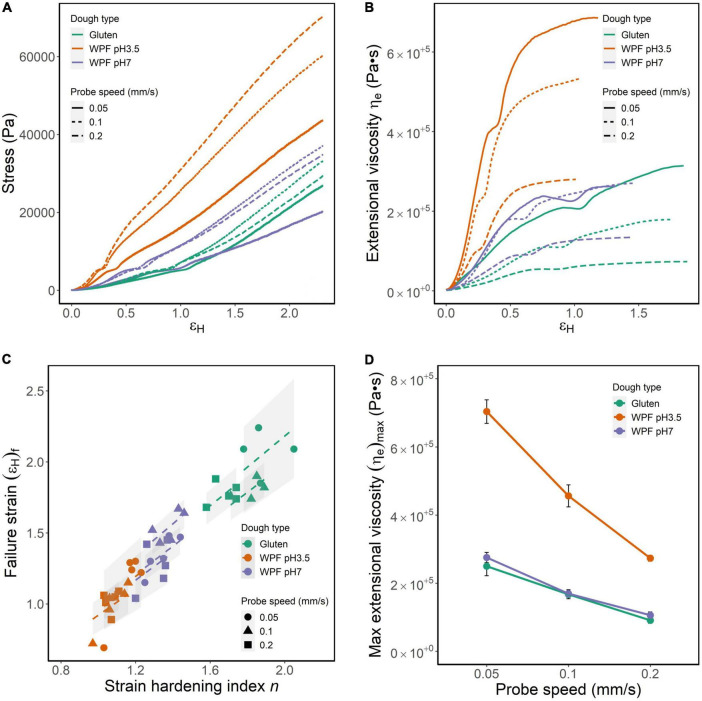
Strain-hardening parameters of gluten, WPF pH3.5, and pH 7 doughs tested by lubricated squeezing flow method with constant probe velocities of 0.05, 0.1, and 0.2 mm/s. **(A)** Representative stress–strain curves. **(B)** Representative curves of stress versus extensional viscosity (η_e_). **(C)** Dough failure strain [(ε_H_)_f_] versus strain-hardening index (n). **(D)** The maximum extensional viscosity during the test as functions of the probe speed. Symbols represent the mean value of (η_e_)_max_, and error bars are calculated standard errors.


The lubricated squeezing flow method with constant probe speeds of 0.05, 0.1, and 0.2 mm/s was performed on gluten, WPF pH3.5, and WPF pH7 doughs. Representative curves of stress (σ) versus Hencky strain (ε_H_) are shown in Figure 6A. Because the tests were performed with a constant probe speed but not controlled Hencky strain rate, the Hencky strain rate varies with the sample height and increases with time in a hyperbolic matter. Thus, it is difficult to plot results in average curves, and a representative curve of each sample under each probe speed is presented instead. An abrupt upturn is observed around the same level of stress in all the curves, which might be caused by the necking of the experimental specimen. Under the biaxial extension, the material necking is often attributed to the imperfection of the material geometry, and for the doughs, which are the biological materials investigated in this study, the inhomogeneity in the cylindrical specimen for the test was hardly evitable (64, 65). Similar observation in log(σ) – log(ε_H_) plots has been explained in a previous study, and it was pointed out that the strain where the turning occurred was usually constant for the same type of polymer (66). An unstable necking would cumulatively increase and eventually lead to the failure of the dough material (67). Despite the existence of the upturn, the stress increased with strain in all the tests, and the curves were individually fitted to a power-law equation as shown below (21):

(6) $$\sigma =K\,\epsilon_{\text{H}}^{n}$$

Where σ is stress (Pa), ε_H_ is Hencky strain, *n* is a strain-hardening index, and *K* is a constant. The adjusted R^2^ of the fitting was in the range of 0.9745–0.9995. The greater value of *n* indicates the greater strain-hardening behavior of the material. *n* values of the doughs under different probe speeds are shown in Figure 6C along the *x*-axis. Generally, the *n* values (mean ± standard deviation) of the dough types were gluten dough (1.79 ± 0.123) >WPF pH7 dough (1.34 ± 0.075) >WPF pH3.5 dough (1.10 ± 0.075), and the differences between dough types were significant (*p* ≤ 0.01). The effect of probe speed on the *n* values was not significant for the WPF pH7 dough as observed in the other two dough types. However, *n* tested at 0.05 and 0.1 mm/s was not significantly different for gluten dough, and at 0.1 and 0.2 mm/s for WPF pH3.5 dough. Unlike the WPF pH3.5 dough, the strain-hardening behavior of WPF pH7 dough was similar to that of gluten dough.

The relationship between σ and ε_H_ was converted to an extensional viscosity (η_e_) versus ε_H_ relationship following Eq. (3). The ε_H_ value when the η_e_ reached a maximum value was defined as failure strain [(ε_H_)_f_]. The (ε_H_)_f_ of each test is shown in Figure 6C. Same as *n*, the values of (ε_H_)_f_ (mean ± standard deviation) followed the order: gluten dough (1.88 ± 0.167) >WPF pH7 dough (1.39 ± 0.176) >WPF pH3.5 dough (1.05 ± 0.183). Due to the variabilities in (ε_H_)_f_ among tests, representative curves instead of average curves before reaching (ε_H_)_f_ for each dough type are illustrated in Figure 6B. With the increase of ε_H_, η_e_ increased despite the dough type and probe speed. The increase of η_e_ was relatively rapid in the lower ε_H_ region, but started to reach an equilibrium approaching (ε_H_)_f_. Figure 6A illustrates a “dip” in η_e_ in all the curves at the ε_H_ where the upturn occurred, which may indicate a localized thinning during the necking of the test specimen. It is shown in Figure 6B that the η_e_ of WPF pH3.5 dough was prominently higher than that of gluten and WPF pH7 dough during the increase of ε_H_. The maxima η_e_, (η_e_)_max_, of all the dough types at different probe speeds are shown in Figure 6D. The values of (η_e_)_max_ decreased with the increase of probe speed, and (η_e_)_max_ value of WPF pH3.5 dough was significantly lower than those for gluten and WPF pH7 doughs regardless of the probe speed (*p* ≤ 0.0001), while there was no significant difference between (η_e_)_max_ of gluten and WPF pH3.5 dough.

The η_e_ measures the resistance to the gas cell growth, and for bread doughs with desirable baking performance, the dough matrix containing the expanding gas cells should be relatively resistant to premature rupture for a better gas retention result (42, 68). However, excessively high η_e_ values may lead to the failure of gas bubble inflation inside the dough matrix, resulting in ineffective gas retention.

A linear relationship between *n* and (ε_H_)_f_ was fitted separately for each group of dough type and probe speed [adjusted *R*^2^ = (0.974, 0.998)] and is shown in Figure 6C as dashed lines. The slopes of the fitted models are in the interval (0.921, 1.090), indicating that a higher *n* value representing a stronger strain-hardening behavior would lead to a greater strain where the ultimate instability within the material occurs. WPF pH3.5 dough showed both lower *n* and lower ε_H_, suggesting that the dough may show less extensibility before structural collapse occurs during extension. Conversely, values of *n* and ε_H_ for the WPF pH7 dough are closer to those of gluten dough. Accordingly, a more extended and thinner dough film between gas cells would be more resistant to further thinning than a less extended and thicker dough film (20). Based on the previous analysis, the dough film of the WPF pH3.5 dough might be thicker due to the retardation of gas cell growth caused by its high extensional viscosity. Therefore, in principle, the WPF pH7 dough may show a baking performance more similar to gluten dough, compared with the WPF pH3.5 dough.

### Microstructure of doughs

**FIGURE 7:**
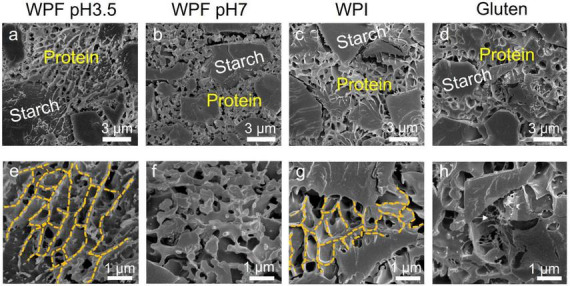
Cryo-scanning electron micrographs of doughs prepared with different proteins. **(a,e)** Whey protein fibril at pH 3.5 (WPF pH3.5). **(b,f)** Whey protein fibril at pH 7 (WPF pH7). **(c,g)** Native whey protein isolate (WPI). **(d,h)** Gluten. Protein and starch in the doughs are labeled with yellow and white texts, respectively. The yellow dashed lines in **(e,g)** represented the network structure formed within WPF pH3.5 and WPI areas. The white arrow in **(h)** highlighted the gluten strands.

Cryo-scanning electron micrographs of the doughs prepared with the different protein forms are illustrated in Figure 7. A continuous protein phase and embedding starch granules with a size of around 3 μm are observed in Figures 7a–d. As discussed in section Introduction, the existence of a protein phase may have contributed to improving the dough cohesiveness compared to the dough prepared without proteins. At higher magnification (Figures 7e–h), WPF pH3.5 formed a porous dough with an interlinked network, and WPF pH7 formed dough with the presence of clusters instead of a visible network structure. Similar structures of WPF have been observed in emulsions stabilized by whey protein fibrils, where oil droplets were surrounded by fibrillar network structures at lower pH and by aggregated structures at higher pH (69). A porous network structure was also identified in the dough prepared with WPI (Figure 7h).

It has been reported in a previous study that the addition of whey protein isolates to a rice flour-based dough formula significantly decreased the storage modulus measured in a frequency sweep test, which is similar to the findings of this study (70). The results indicated that the existence of a protein network structure did not necessarily guarantee good material strength. The improved rheological performances of WPFs compared with native WPI may be resulted from the enhanced interfacial properties related to fibril length, and longer fibrils could form polar–non-polar interfaces resulting in higher interfacial moduli, which is absent when native WPI is used (37).

The structure of the gluten dough prepared with gluten and non-wheat starch (Figure 7h) showed similar properties to those prepared from wheat flour, which has been demonstrated extensively in the literature (71–73). Note that besides the evident gluten matrix, gluten strands (indicated by the white arrow) may also positively contribute to the rheological properties and baking performance of the dough. It has been proposed that the gluten strands are usually in an un-extended state with loops and knots formed by the random arrangement of the protein. In another study, it was stated that the elimination of sodium chloride in dough mixing altered the gluten structure from elongated strains to less connected particles, resulting in rheological properties unfavorable for good baking performance (74, 75). The capability of forming strands may be another factor that needs to be taken into consideration during the search for a high-quality gluten replacer.

## Conclusion

In this study, whey protein fibrils prepared at pH 3.5 and pH 7 were incorporated into a gluten-free formulated dough. The doughs showed comparable linear and non-linear rheological properties to gluten dough, particularly the dough prepared with fibrils produced at pH 7. The pH 7 whey protein fibrils enabled the dough to have similar creep recovery behavior in the non-linear viscoelastic region and strain-hardening properties to gluten dough.

The rheological properties of the dough were related to the microstructure of the protein phase, where fibrils at pH 7 formed aggregates, while fibrils at pH 3.5 formed an interlinked, porous network.

It can be concluded that whey protein fibrils could be used as a gluten replacer in the development of gluten-free baked products. As the first attempt at incorporating whey protein fibrils into a gluten-free dough, the study focused on the effects of protein on the rheological properties. The rheological properties could also be affected by the interactions between protein fibrils and starch, of which the mechanism of the interaction remains unclear and should be assessed in future studies. Additionally, the ingestion of protein fibrils may raise health concerns due to its structural resemblance to disease-related amyloids. Although fibrils derived from food proteins are generally considered safe, a thorough investigation of the cytotoxicity should be performed before the application of the fibrils in food products to eliminate ingestion risks (28).

## Data availability statement

The original contributions presented in this study are included in the article/supplementary material, further inquiries can be directed to the corresponding authors.

## Author contributions

SS: conceptualization, methodology, formal analysis, investigation, and writing – original draft. DC: conceptualization, methodology, formal analysis, investigation, and reviewing and editing of the manuscript. EF: formal analysis and investigation. OJ: conceptualization, resources, supervision, project administration, and funding acquisition. OC: conceptualization, resources, supervision, reviewing and editing, project administration, and funding acquisition. All authors contributed to the article and approved the submitted version.

## References

[1] Green PHR Cellier C. Celiac disease. *N Engl J Med.* (2007) 357:1731–43. 10.1056/NEJMra071600 17960014

[2] Saturni L Ferretti G Bacchetti T. The gluten-free diet: safety and nutritional quality. *Nutrients.* (2010) 2:16–34. 10.3390/nu2010016 22253989 PMC3257612

[3] RodrigoL. Celiac disease. *World J Gastroenterol.* (2006) 12:6577. 10.3748/wjg.v12.i41.6585 17075969

[4] Reilly NR. The gluten-free diet: recognizing fact, fiction, and fad. *J Pediatr.* (2016) 175:206–10. 10.1016/j.jpeds.2016.04.014 27185419

[5] Shan L Molberg Ø Parrot I Hausch F Filiz F Gray GM Structural basis for gluten intolerance in celiac sprue. *Science.* (2002) 297:2275–9. 10.1126/science.1074129 12351792

[6] Day L Augustin MA Batey IL Wrigley CW. Wheat-gluten uses and industry needs. *Trends food Sci Technol.* (2006) 17:82–90. 10.1016/j.tifs.2005.10.003

[7] Wieser H. Chemistry of gluten proteins. *Food Microbiol.* (2007) 24:115–9. 10.1016/j.fm.2006.07.004 17008153

[8] Veraverbeke WS Delcour JA. Wheat protein composition and properties of wheat glutenin in relation to breadmaking functionality. *Crit Rev Food Sci Nutr.* (2002) 42:179–208. 10.1080/10408690290825510 12058979

[9] Delcour JA Joye IJ Pareyt B Wilderjans E Brijs K Lagrain B. Wheat gluten functionality as a quality determinant in cereal-based food products. *Annu Rev Food Sci Technol.* (2012) 3:469–92. 10.1146/annurev-food-022811-101303 22224557

[10] Naqash F Gani A Gani A Masoodi FA. Gluten-free baking: combating the challenges-a review. *Trends Food Sci Technol.* (2017) 66:98–107. 10.1016/j.tifs.2017.06.004

[11] Capriles VD Arêas JAG. Novel approaches in gluten-free breadmaking: interface between food science, nutrition, and health. *Compr Rev Food Sci Food Saf.* (2014) 13:871–90. 10.1111/1541-4337.12091

[12] Houben A Höchstötter A Becker T. Possibilities to increase the quality in gluten-free bread production: an overview. *Eur Food Res Technol.* (2012) 235:195–208. 10.1007/s00217-012-1720-0

[13] Hibberd GE Wallace WJ. Dynamic viscoelastic behaviour of wheat flour doughs. *Rheol Acta.* (1966) 5:193–8. 10.1007/BF01982426

[14] Phan-Thien N Safari-Ardi M Morales-Patiño A. Oscillatory and simple shear flows of a flour-water dough: a constitutive model. *Rheol Acta.* (1997) 36:38–48. 10.1007/BF00366722

[15] Edwards NM Dexter JE Scanlon MG. Starch participation in durum dough linear viscoelastic properties. *Cereal Chem.* (2002) 79:850–6. 10.1094/CCHEM.2002.79.6.850

[16] Rosell CM Foegeding A. Interaction of hydroxypropylmethylcellulose with gluten proteins: small deformation properties during thermal treatment. *Food Hydrocoll.* (2007) 21:1092–100. 10.1016/j.foodhyd.2006.08.003

[17] Li W Dobraszczyk BJ Schofield JD. Stress relaxation behavior of wheat dough, gluten, and gluten protein fractions. *Cereal Chem.* (2003) 80:333–8. 10.1094/CCHEM.2003.80.3.333

[18] Dobraszczyk BJ Morgenstern MP. Rheology and the breadmaking process. *J Cereal Sci.* (2003) 38:229–45. 10.1016/S0733-5210(03)00059-6

[19] Lefebvre J. An outline of the non-linear viscoelastic behaviour of wheat flour dough in shear. *Rheol Acta.* (2006) 45:525–38. 10.1007/s00397-006-0093-3

[20] Van Vliet T Janssen AM Bloksma AH Walstra P. Strain hardening of dough as a requirement for gas retention. *J Texture Stud.* (1992) 23:439–60. 10.1111/j.1745-4603.1992.tb00033.x

[21] Dobraszczyk BJ Roberts CA. Strain hardening and dough gas cell-wall failure in biaxial extension. *J Cereal Sci.* (1994) 20:265–74. 10.1006/jcrs.1994.1066

[22] Anton AA Artfield SD. Hydrocolloids in gluten-free breads: a review. *Int J Food Sci Nutr.* (2008) 59:11–23. 10.1080/09637480701625630 18097842

[23] Matos ME Sanz T Rosell CM. Establishing the function of proteins on the rheological and quality properties of rice based gluten free muffins. *Food Hydrocoll.* (2014) 35:150–8. 10.1016/j.foodhyd.2013.05.007

[24] Rosell CM. Enzymatic manipulation of gluten-free breads. In: Gallagher E editor. *Gluten-Free Food Science and Technology.* Chichester: Wiley-Blackwell (2009). p. 83–98. 10.1002/9781444316209.ch6

[25] Arendt EK Morrissey A Moore MM Dal Bello F. Gluten-free breads. In: Arendt EK Dal Bello F editors. *Gluten-Free Cereal Products and Beverages*. Amsterdam: Elsevier (2008). p. 289–319. 10.10I6/B978-012373739-7.500I5-0

[26] Moore MM Heinbockel M Dockery P Ulmer HM Arendt EK. Network formation in gluten-free bread with application of transglutaminase. *Cereal Chem.* (2006) 83:28–36. 10.1094/CC-83-0028

[27] van Riemsdijk LE van der Goot AJ Hamer RJ Boom RM. Preparation of gluten-free bread using a meso-structured whey protein particle system. *J Cereal Sci.* (2011) 53:355–61. 10.1016/j.jcs.2011.02.006

[28] Cao Y Mezzenga R. Food protein amyloid fibrils: origin, structure, formation, characterization, applications and health implications. *Adv Colloid Interface Sci.* (2019) 269:334–56. 10.1016/j.cis.2019.05.002 31128463

[29] Sun C Wang C Xiong Z Fang Y. Properties of binary complexes of whey protein fibril and gum arabic and their functions of stabilizing emulsions and simulating mayonnaise. *Innov Food Sci Emerg Technol.* (2021) 68:102609. 10.1016/j.ifset.2021.102609

[30] Chen D Narayanan N Federici E Yang Z Zuo X Gao J Electrospinning induced orientation of protein fibrils. *Biomacromolecules.* (2020) 21:2772–85. 10.1021/acs.biomac.0c00500 32463660

[31] Mantovani RA de Figueiredo Furtado G Netto FM Cunha RL. Assessing the potential of whey protein fibril as emulsifier. *J Food Eng.* (2018) 223:99–108. 10.1016/j.jfoodeng.2017.12.006

[32] Mohammadian M Madadlou A. Characterization of fibrillated antioxidant whey protein hydrolysate and comparison with fibrillated protein solution. *Food Hydrocoll.* (2016) 52:221–30. 10.1016/j.foodhyd.2015.06.022

[33] Bolder SG Vasbinder AJ Sagis LMC van der Linden E. Heat-induced whey protein isolate fibrils: conversion, hydrolysis, and disulphide bond formation. *Int Dairy J.* (2007) 17:846–53. 10.1016/j.idairyj.2006.10.002

[34] der Goot AJ Peighambardoust SH Akkermans C van Oosten-Manski JM. Creating novel structures in food materials: the role of well-defined shear flow. *Food Biophys.* (2008) 3:120–5. 10.1007/s11483-008-9081-8

[35] Adamcik J Berquand A Mezzenga R. Single-step direct measurement of amyloid fibrils stiffness by peak force quantitative nanomechanical atomic force microscopy. *Appl Phys Lett.* (2011) 98:193701. 10.1063/1.3589369

[36] Peng D Yang J Li J Tang C Li B. Foams stabilized by β-lactoglobulin amyloid fibrils: effect of pH. *J Agric Food Chem.* (2017) 65:10658–65. 10.1021/acs.jafc.7b03669 29135243

[37] Jung J-M Gunes DZ Mezzenga R. Interfacial activity and interfacial shear rheology of native β-lactoglobulin monomers and their heat-induced fibers. *Langmuir.* (2010) 26:15366–75. 10.1021/la102721m 20825171

[38] Chen D Fang F Federici E Campanella O Jones OG. Rheology, microstructure and phase behavior of potato starch-protein fibril mixed gel. *Carbohydr Polym.* (2020) 239:116247. 10.1016/j.carbpol.2020.116247 32414456

[39] Kroes-Nijboer A Sawalha H Venema P Bot A Flöter E den Adel R Stability of aqueous food grade fibrillar systems against pH change. *Faraday Discuss.* (2012) 158:125–38. 10.1039/c2fd20031g 23234164

[40] Campanella OH. Instrumental techniques for measurement of textural and rheological properties of foods. In: Cho Y-J Kang S editors. *Emerging Technologies for Food Quality and Food Safety Evaluation*. New York, NY: CRC Press Taylor and Francis Group (2011). p. 6–54. 10.1201/b10710-3

[41] Tarhan O Spotti MJ Schaffter S Corvalan CM Campanella OH. Rheological and structural characterization of whey protein gelation induced by enzymatic hydrolysis. *Food Hydrocoll.* (2016) 61:211–20. 10.1016/j.foodhyd.2016.04.042

[42] Kokelaar JJ Van Vliet T Prins A. Strain hardening properties and extensibility of flour and gluten doughs in relation to breadmaking performance. *J Cereal Sci.* (1996) 24:199–214. 10.1006/jcrs.1996.0053

[43] Campanella OH Peleg M. Squeezing flow viscometry for nonelastic semiliquid foods—theory and applications. *Crit Rev Food Sci Nutr.* (2002) 42:241–64. 10.1080/10408690290825547 12058982

[44] Akkermans C van der Goot AJ Venema P van der Linden E Boom RM. Formation of fibrillar whey protein aggregates: influence of heat and shear treatment, and resulting rheology. *Food Hydrocoll.* (2008) 22:1315–25. 10.1016/j.foodhyd.2007.07.001

[45] Loveday SM Su J Rao MA Anema SG Singh H. Effect of calcium on the morphology and functionality of whey protein nanofibrils. *Biomacromolecules.* (2011) 12:3780–8. 10.1021/bm201013b 21894942

[46] Bolder SG Sagis LMC Venema P van der Linden E. Effect of stirring and seeding on whey protein fibril formation. *J Agric Food Chem.* (2007) 55:5661–9. 10.1021/jf063351r 17571895

[47] Morrison FA. Material functions. In: *Understanding Rheology*. New York, NY: Oxford University Press (2001). p. 131–68. Available online at: https://global.oup.com/ushe/product/understanding-rheology-9780195141665?cc=us&lang=en&#:~:text=Understanding%20Rheology%20incorporates%20helpful%20pedagogical,of%20constitutive%20equations%2C%20and%20birefringence

[48] Hyun K Kim SH Ahn KH Lee SJ. Large amplitude oscillatory shear as a way to classify the complex fluids. *J Nonnewton Fluid Mech.* (2002) 107:51–65. 10.1016/S0377-0257(02)00141-6

[49] Cho KS Hyun K Ahn KH Lee SJ. A geometrical interpretation of large amplitude oscillatory shear response. *J Rheol.* (2005) 49:747–58. 10.1122/1.1895801

[50] Pole SS Isayev AI. Correlations in rheological behavior between large amplitude oscillatory shear and steady shear flow of silica-filled star-shaped styrene-butadiene rubber compounds: experiment and simulation. *J Appl Polym Sci.* (2021) 138:50660. 10.1002/app.50660

[51] Autio K Flander L Kinnunen A Heinonen R. Bread quality relationship with rheological measurements of wheat flour dough. *Cereal Chem.* (2001) 78:654–7. 10.1094/CCHEM.2001.78.6.654

[52] Stojceska V Butler F Gallagher E Keehan D. A comparison of the ability of several small and large deformation rheological measurements of wheat dough to predict baking behaviour. *J Food Eng.* (2007) 83:475–82. 10.1016/j.jfoodeng.2007.02.043

[53] Zare Y Park SP Rhee KY. Analysis of complex viscosity and shear thinning behavior in poly (lactic acid)/poly (ethylene oxide)/carbon nanotubes biosensor based on Carreau–Yasuda model. *Results Phys.* (2019) 13:102245. 10.1016/j.rinp.2019.102245

[54] Wachsstock DH Schwarz WH Pollard TD. Cross-linker dynamics determine the mechanical properties of actin gels. *Biophys J.* (1994) 66:801–9. 10.1016/S0006-3495(94)80856-2 8011912 PMC1275778

[55] Akkermans C der Goot AJ Venema P der Linden E Boom RM. Properties of protein fibrils in whey protein isolate solutions: microstructure, flow behaviour and gelation. *Int Dairy J.* (2008) 18:1034–42. 10.1016/j.idairyj.2008.05.006

[56] Liu Y Liu D Wei G Ma Y Bhandari B Zhou P. 3D printed milk protein food simulant: improving the printing performance of milk protein concentration by incorporating whey protein isolate. *Innov Food Sci Emerg Technol.* (2018) 49:116–26. 10.1016/j.ifset.2018.07.018

[57] Chow CY Thybo CD Sager VF Riantiningtyas RR Bredie WLP Ahrné L. Printability, stability and sensory properties of protein-enriched 3D-printed lemon mousse for personalised in-between meals. *Food Hydrocoll.* (2021) 120:106943. 10.1016/j.foodhyd.2021.106943

[58] Ni S Jiao L Zhang H Zhang Y Fang G Xiao H Enhancing hydrophobicity, strength and UV shielding capacity of starch film via novel co-cross-linking in neutral conditions. *R Soc open Sci.* (2018) 5:181206. 10.1098/rsos.181206 30564411 PMC6281899

[59] Zuo Y He X Li P Li W Wu Y. Preparation and characterization of hydrophobically grafted starches by in situ solid phase polymerization. *Polymers (Basel).* (2019) 11:72. 10.3390/polym11010072 30960056 PMC6402000

[60] Tweedie CA Van Vliet KJ. Contact creep compliance of viscoelastic materials via nanoindentation. *J Mater Res.* (2006) 21:1576–89. 10.1557/jmr.2006.0197

[61] Khatkar BS Schofield JD. Dynamic rheology of wheat flour dough. II. Assessment of dough strength and bread-making quality. *J Sci Food Agric.* (2002) 82:823–6. 10.1002/jsfa.1111

[62] Edwards NM Dexter JE Scanlon MG Cenkowski S. Relationship of creep-recovery and dynamic oscillatory measurements to durum wheat physical dough properties. *Cereal Chem.* (1999) 76:638–45. 10.1094/CCHEM.1999.76.5.638

[63] Safari-Ardi M Phan-Thien N. Stress relaxation and oscillatory tests to distinguish between doughs prepared from wheat flours of different varietal origin. *Cereal Chem.* (1998) 75:80–4. 10.1094/CCHEM.1998.75.1.80

[64] Li M Chandra A. Influence of strain-rate sensitivity on necking and instability in sheet metal forming. *J Mater Process Technol.* (1999) 96:133–8. 10.1016/S0924-0136(99)00321-0

[65] Tvergaard V. Effect of kinematic hardening on localized necking in biaxially stretched sheets. *Int J Mech Sci.* (1978) 20:651–8. 10.1016/0020-7403(78)90023-1

[66] Wikström K Bohlin L. Extensional flow studies of wheat flour dough. II. Experimental method for measurements in constant extension rate squeezing flow and application to flours varying in breadmaking performance. *J Cereal Sci.* (1999) 29:227–34. 10.1006/jcrs.1999.0252

[67] Dobraszczyk BJ. The physics of baking: rheological and polymer molecular structure–function relationships in breadmaking. *J Nonnewton Fluid Mech.* (2004) 124:61–9. 10.1016/j.jnnfm.2004.07.014

[68] Rouillé J Della Valle G Lefebvre J Sliwinski E vanVliet T. Shear and extensional properties of bread doughs affected by their minor components. *J Cereal Sci.* (2005) 42:45–57. 10.1016/j.jcs.2004.12.008

[69] Cui F McClements DJ Liu X Liu F Ngai T. Development of pH-responsive emulsions stabilized by whey protein fibrils. *Food Hydrocoll.* (2022) 122:107067. 10.1016/j.foodhyd.2021.107067

[70] Marcoa C Rosell CM. Effect of different protein isolates and transglutaminase on rice flour properties. *J Food Eng.* (2008) 84:132–9. 10.1016/j.jfoodeng.2007.05.003

[71] Amend T Belitz H-D. The formation of dough and gluten-a study by scanning electron microscopy. *Zeitschrift für Leb Forsch.* (1990) 190:401–9. 10.1007/BF01202557

[72] Esselink E Van Aalst H Maliepaard M Henderson TMH Hoekstra NLL van Duynhoven J. Impact of industrial dough processing on structure: a rheology, nuclear magnetic resonance, and electron microscopy study. *Cereal Chem.* (2003) 80:419–23. 10.1094/CCHEM.2003.80.4.419

[73] Khatkar BS Barak S Mudgil D. Effects of gliadin addition on the rheological, microscopic and thermal characteristics of wheat gluten. *Int J Biol Macromol.* (2013) 53:38–41. 10.1016/j.ijbiomac.2012.11.002 23142154

[74] Amend T Belitz H-D. Microstructural studies of gluten and a hypothesis on dough formation. *Food Struct.* (1991) 10:1.

[75] Beck M Jekle M Becker T. Impact of sodium chloride on wheat flour dough for yeast-leavened products. I. Rheological attributes. *J Sci Food Agric.* (2012) 92:585–92. 21953245 10.1002/jsfa.4612

